# Examining total and domain-specific sedentary behaviour using the socio-ecological model – a cross-sectional study of Irish adults

**DOI:** 10.1186/s12889-019-7447-0

**Published:** 2019-08-22

**Authors:** Gail Nicolson, Catherine Hayes, Catherine Darker

**Affiliations:** Public Health & Primary Care, Trinity College Dublin, Institute of Population Health, Russell Centre, Tallaght Cross, D24 DH74 Dublin, Ireland

**Keywords:** Sitting, Sedentary behaviour, Adults, Ecological model, Correlates, Intrapersonal, Interpersonal, Environment, Occupational, Screen-time, Transportation, Leisure

## Abstract

**Background:**

Sedentary behaviour (SB) has been linked with detrimental effects on morbidity and mortality. This study aims to identify the individual, social and environmental correlates of total sedentary behaviour and the contexts in which sitting time accumulates in an Irish adult cohort.

**Methods:**

Cross-sectional analysis of data from 7328 adults of the nationally representative Healthy Ireland Survey. Ordinal regression analyses were used to examine participants’ socio-demographic characteristics, lifestyle factors, physical and mental health status, perceived neighbourhood environmental factors, and their association with total daily sitting times and sitting times across the domains of occupation, leisure screen-time and transportation/leisure.

**Results:**

Overall median of sitting time per day was 450 min (7.5 h). Male gender, and living in an urban location were associated with increased total-, occupational, and screen-time sitting (*p* < 0.001). Younger age was associated with increased total and occupational sitting times (*p* < 0.001), while being older was associated with increased screen-time and transportation/leisure sitting (*p* < 0.001). Insufficient physical activity levels were associated with increased sitting across all domains (*p* < 0.001). Higher socio-economic classification and education levels were associated with increased total, occupational, and transportation/leisure SB (*p* < 0.001), while lower socio-economic classification and education levels were associated with increased screen-time sitting (p < 0.001). Alcohol consumption was associated with screen-time and transportation/leisure sitting (*p* < 0.01), while smoking was associated with increased screen-time sitting (*p* < 0.001). Being married was associated with less screen-time (p < 0.001) and transportation/leisure sitting (*p* = 0.02), while those with a caring role had less total (*p* = 0.04) and screen-time sitting (*p* = 0.01). A significant negative association between neighbourhood attributes and total (p = 0.04), and transportation/leisure sitting times (*p* < 0.001) was found.

**Conclusion:**

The results of this study provide a starting position for development of targeted interventions aimed at the most sedentary, such as males with sedentary occupations in higher socio-economic groups and education levels, those with insufficient levels of physical activity and who live in an urban location.

## Background

Insufficient physical activity is defined as less than 150 min of moderate-intensity physical activity per week, or less than 75 min of vigorous-intensity physical activity per week, or equivalent [1]. At a global level, one in four adults are not sufficiently active [1]. Ireland has low levels of physical activity (PA) among adults in Europe (ranked 30th of 44 countries) [2], and just one third (32.6%) of Irish adults achieve the minimum level of activity of 150 min of moderate physical activity per week set by the National Physical Activity Guidelines [3]. Instead, people spend the majority of their time being sedentary [4, 5]. Sedentary behaviour (SB) is defined as any behaviour characterised by an energy expenditure of ≤1.5 metabolic equivalents (METs), while awake, and in a sitting, reclining or lying posture and is distinct from a simple absence of physical activity [6, 7]. SB has been reported to be a risk factor for a host of adverse health outcomes including the development of chronic diseases such as depression [8], type 2 diabetes [9], cardiovascular disease [10, 11], osteoporosis [12], some cancers [13, 14], as well as premature death and overall mortality [13, 15]. It has been estimated that having taken physical activity into account, adults who sit for more than 10 h per day have a 34% higher all-cause mortality risk; the risk appears to increase significantly when sitting exceeds 7 h per day [16]. To negate the risks associated with high levels of sitting, daily physical activity must exceed current recommendations of ≥ 60–75 min of moderate physical activity per day; however those who achieve this level represent a very small proportion of adults [17]. Research into the prevalence and determinants of SB to identify the populations most at risk with longest sitting times, to explore the contexts in which most SB occurs.

The focus to date on factors that influence SB has mostly been directed at individual level factors such as biological, psychological and behavioural [18, 19], or socio-demographic factors in isolation with more distal contextual factors such as the built, social and economic environment overlooked in many studies [20]. The socio-ecological theory of health behaviour recognises that individual behaviours operate in, and are affected by environmental and policy contexts [21]. This conceptualisation of SB leads to explicit consideration of complex multiple levels of influence, i.e. intrapersonal (biological, psychological), interpersonal (social, cultural), organisational, community, physical environment, and policy [18]. The socio-ecological model (SEM) posits that simple cause and effect pathways of health behaviours are unlikely, and motivating or educating an individual to change their behaviour is likely to be restricted if their physical and socio-cultural environments do not enable and support the behaviour [22]. The SEM places the individual at the centre of an ecosystem, and provides a useful and integrative framework to achieve better understanding of the multiple factors and barriers that impact SB. Central to the SEM, the ‘behaviour settings’ construct highlights the influence of particular contexts or domains in which behaviours occur [23]. Owen et al. [18] not only advocate this model of SB to understand the correlates of time spent sitting across different domains of leisure, transportation, and occupation, but also the necessity to identify and understand modifiable factors within these settings, to develop effective interventions and appropriate policies to address these. Early research on SB focussed heavily on TV viewing, however SB accumulates across many contexts during waking hours, including the workplace, transportation and domestic environments [18]. Various factors are likely to influence an individual’s choice and/or risk of engaging in SBs, while built environments and social norms may encourage and reinforce time spent sitting [18]. Knowledge about the various levels and types of influences and contributors to SB may inform the development of multi-level interventions that offer an optimum level of success [21].

Systematic reviews assessing the available evidence on socio-ecological factors influencing SB across the life course; in youth [24], those aged 18–65 [19], and older adults [25] have provided information to map the domains of SB, as well as a conceptual approach to understand determinants of prolonged sitting time. Older females with low levels of physical activity, higher body mass index, who smoke and consume high levels of snack foods have higher total and leisure sitting times, whereas SB in the context of transport has been found greatest in higher income males [19]. These findings emphasise the necessity to focus on separate domains of SB. Socio-economic status is indicated to be the most consistent factor of all of the individual level factors associated with television viewing SB and occupational SB [19]. A recent review by Prince et al. [26] identified individual-level correlates; biological (i.e. age, gender, body composition and health status), behavioural (i.e. lifestyle, physical activity and sedentary habits), psychological (i.e. stress, mental health, attitudes and perceptions), and socio-economic factors (education level, employment status, and income) as important correlates of SB. The authors state that despite calls for the use of the SEM approach to look at determinants of SB, intrapersonal factors are the focus in the majority of studies investigating SB. Interpersonal factors such as marital status [27, 28], and family and caring duties [29] may be potential correlates associated with sitting time. At an environmental level, correlates of SB include physical environment and neighbourhood attributes such as safety and walkability [19]. Inconsistent and mixed results have been reported on the association between SB and perceived neighbourhood attributes such as open spaces [19]. Neighbourhood aesthetics have been found to be associated with overall sitting times [28] and women in neighbourhoods with high walkability have been found to spend less time watching TV [30]. A correlation between living in an urban location and longer sitting times has been found in some studies [29, 31]. In a recent systematic review investigating the association between physical environment and weight status in adults [32], urban sprawl and land use mix, were found to influence weight status in the US only. Busschaert et al. [33] used a range of socio-ecological factors related to context-specific sitting times. However, this was a small (*n* = 301) cohort and the physical environment correlates used in the study focussed particularly on the close proximal environment. The neighbourhood environment access and characteristics as identified by Owen et al. [18] and the SEM, such as perceived aesthetics and open space availability that may influence SB [34] are also important to understanding SB. The present study includes a range of perceived neighbourhood attributes that may be associated with SB in a large generalisable sample, thereby adding to the knowledge regarding environmental factors as identified in the SEM.

**Fig. 1:**
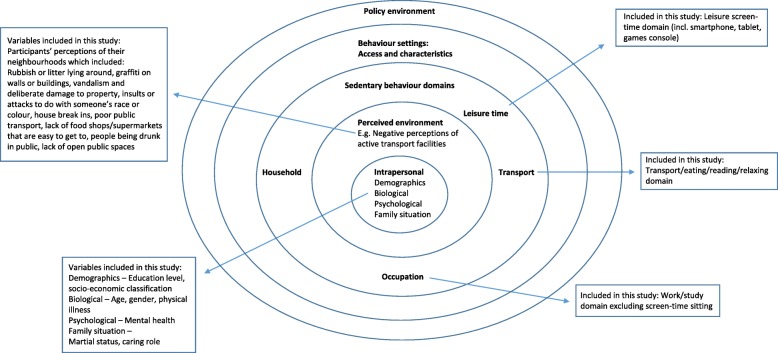
Mapping of the domains and correlates available in the current study to the socio-ecological model (Adapted with permission from Owen et al., 2011 [18])

One of the research priorities identified in Owen et al. (2011) is to gather evidence on all of the levels of influence on SB across different countries where environmental, social and cultural attributes may differ, to allow for the characterisation of a broader range of variation in individual, social and environmental correlates. Differences in sitting times have been reported in European contexts [35], in the US [36] and in Australia [37],whereas Matthews et al. [36] used objectively measured sitting time however contextual information on the domains in which the patterns of accumulated sedentariness occured were not reported. An understanding of socio-ecological factors at each level, that are most relevant to specific populations, and how these factors may relate to each other in SB, is necessary if SB is to be successfully targeted in interventions. Loyen et al. [35] used the International Physical Activity Questionnaire (IPAQ) to determine SB. A breakdown of the separate domains in which sitting occurs was not used. In a study of Australian adults' sitting, [37] only two domains (occupational and leisure-time sitting) were assessed – other domains in which SB accumulates such as transport-related sitting were not included [38]. More accurate measures of total daily SB include the key domains that contribute to total sitting time: work, screen-time, leisure-time and transportation SB [18, 39]. TV viewing and occupational sitting time contribute to the majority of total amount of sitting accumulated throughout the day [40]. In recent years, mobile devices have enabled consumers to watch television programming at any time and location [41]. The use of smartphones and tablets, together with the streaming services have changed the way audiences view programmes. Transportation SB is the context in which the least amount of daily SB accumulates, 60 min per day compared with 390 min and 120 min of occupational and TV viewing SB respectively [42], within the three domains used in the SEM. Television viewing has been found to be directly associated with all-cause mortality whereas time spent driving was not significantly associated with higher mortality in a large (*n* = 13,284) cohort of Spanish university graduates [43]. However, the higher educational levels of the participants in this study may have accounted for the lower than expected mortality rate observed; therefore the results may not be generalisable. Stamatakis et al. [44] assessed self-reported SB in the contexts of TV (including DVDs and videos) viewing; and sitting during non-work times, including reading and computer use. Participants who were in employment in this study were also assessed on average daily times spent sitting or standing while at work. Transportation SB was not investigated as a separate domain. It can be argued that although it is necessary to include transportation SB in overall daily measurements of SB, if the objective of interventions is to target the context in which most risk occurs, it may not be necessary to place transportation as a high-risk target for interventions to reduce SB. Transportation SB was included together with other leisure contexts of sitting (reading, relaxing, eating) in the present study. Although transportation SB is a separate domain outlined in the SEM, the data that is available in the present dataset can be usefully applied to the model to highlight sitting correlates in a population-level cohort. See Fig. 1 for a graphical illustration of how the present study has been mapped onto to Owen et al’s [18] SEM.

Although some correlates of SB were examined in the previously mentioned studies [35, 37], it is of value to investigate the intrapersonal (psychological factors, risky health behaviours) and environmental factors (neighbourhood environment) that are emphasised by the SEM together in a large population-level study of adults with a wide-range of ages (18–97 years). The aims of this study were, to compare overall sitting between different individual, social, and environmental categories in a population-level study, and to identify individual, social, and environmental level correlates associated with sitting time across these domain-specific physical and social contexts where most sitting behaviours occur.

## Methods

### Study design

The sample comprised 7328 individuals aged 18 and older participating in the Healthy Ireland Survey (2016) [45]. This nationally representative survey is carried out on an annual basis. Data were collected by the market research company Ipsos MRBI. The provision of access to the data rests with the Irish Department of Health, and is available to researchers fulfilling assessment criteria. The study uses the Irish postal service/Ordinance Survey Ireland’s GeoDirectory as the primary sampling frame [46]. GeoDirectory is a complete database of every building in the Republic of Ireland. A two-stage equal-probability sample of addresses was drawn, and the sample was issued by Electoral Division clusters, each cluster comprising 20 addresses. The initial stage of the sampling process was to select a representative distribution of sampling points around the country. The use of a probability sampling approach ensures that the survey sample comprehensively represents the defined population. In adopting this approach every member of the defined population has a calculable chance of being included in the sample. Individuals in each household were randomly selected using a Kish Grid – a selection process used for random sampling [46]. Fieldwork was conducted between September 2015 and May 2016. Approval to conduct the original study was provided by the Research Ethics Committee of the Royal College of Physicians of Ireland. Informed consent, recorded electronically, was obtained from each participant prior to commencement of the interviews. Data collection was carried out by trained interviewers, and completed on a Computer Assisted Personal Interview (CAPI) basis. Sources, as well as reliability and validity of questionnaire instruments are provided elsewhere [46]. The response rate was 59.9%.

Ethical approval for secondary data analysis was granted by the Research Ethics Committee, School of Medicine, Trinity College Dublin (ref. 20180517).

Variables for analyses were selected a priori, guided by the SEM, and classified according to multiple levels of influence: [intrapersonal] (i) biological and demographic; (ii) psychological and emotional; (iii) behavioural; [interpersonal] (iv) social and cultural; and (v) environment. Variables measuring potential policy or organisational factors that may influence SB were not available in the dataset. This was due to limitations in the scope of the questionnaire given the broad range of topics that are covered in the Healthy Ireland Survey.

### Dependent variable

Sitting time was assessed in minutes using the following measure,

I would now like to ask you a few questions about how much time you spent sitting down yesterday. It may be the case that yesterday was unusual in some way, but it is very important for this study that you answer these questions about yesterday rather than what you might consider to be a normal day:

- Thinking of yesterday, how much time did you spend sitting watching TV or another type of screen such as a computer, tablet, Ipad, smartphone, games console, Kindle etc.? Please do not include any time spent in front of a screen for work or study purposes.
- Thinking again of yesterday, how much time did you spend sitting while engaged in driving, eating, drinking, relaxing, reading etc. Please do not include any time that you already mentioned at the previous question.
- And again thinking of yesterday, how much time did you spend sitting whilst working or studying. Please do not include any time that you already mentioned at the previous questions.

For the current study, total sitting time was calculated by summing the values of (a), (b) and (c).

### Intrapersonal correlates

#### Biological and demographic factors

Respondents provided information about their age, gender and physical health status. Physical health was measured by asking participants if they had any long-standing illness or health problem, i.e. problems which have lasted or will last for at least 6 months or more. Responses were a dichotomous ‘yes’ versus ‘no’.

Socio-demographic variables included education level attained and socio-economic classification level. The original eight level education variable was re-classified for the current study into five simpler categories for ease of analysis (early childhood, primary education, lower secondary; upper secondary; tertiary, post-secondary, non-tertiary; bachelors or equivalent, masters or equivalent, doctoral or equivalent). This variable was dichotomised [early childhood, primary education, lower secondary; upper secondary versus tertiary, post-secondary, non-tertiary; bachelors or equivalent, masters or equivalent, doctoral or equivalent] in the regression models. Socio-economic classification was categorised in four levels (high-managerial, administrative, professional occupation; intermediate occupations; routine and manual occupations; not classified). This was dichotomised (high-managerial, administrative, professional occupation; intermediate occupations versus routine and manual occupations; not classified) for use in the regression models in the present study.

#### Psychological factors

The variable measuring psychological distress recorded the presence or absence of symptoms such as anxiety or depression using the instrument Mental Health Index-5 [74], a subscale of the Short-Form 36 questionnaire (SF-36) [46﻿]. A cut-off point of ≤56 predicts disorder, and this was dichotomised to ‘probable mental health problem’ versus ‘no probable mental health problem’.

#### Behavioural factors

Regarding physical activity, participants were asked, ‘do you think you generally do enough physical activity?’ Dichotomous responses of ‘yes’ versus ‘no’ were used in the analyses. Smoking behaviour was dichotomised in the present study into ‘daily/occasionally’ versus ‘no’. With regard to alcohol consumption, the AUDIT-C is an alcohol-screening tool that can help identify individuals who are hazardous drinkers or have active alcohol use disorders (including alcohol abuse or dependence) [﻿75]. Dangerous alcohol consumption was measured by using questions on drinking behaviour that were scored on a scale of 0–12 (scores of zero reflect no alcohol use) and ranked on the AUDIT-C scale. This was included as a continuous variable in the regression analyses.

#### Interpersonal correlates

Marital status was recoded in the present study into two groups: married or civil partnership; versus single, widowed, divorced, separated. Participants were asked if they provided regular unpaid personal help for a friend or family member with a long-term illness, health problem or disability, to include caring responsibilities as a variable in the analyses. Dichomotised ‘yes’ versus ‘no’ were the response categories.

#### Environmental correlates

Questions regarding participants’ perceptions of their neighbourhoods included whether they thought the following were 'a big problem', 'a bit of a problem' or 'not a problem': rubbish or litter lying around; graffiti on walls or buildings; vandalism and deliberate damage to property; insults or attacks to do with someone’s race or colour; house break ins; poor public transport; lack of food shops/supermarkets that are easy to get to; people being drunk in public; and lack of open public spaces. For the purpose of this analyses, all questions were dichotomised as ‘a big problem’ and ‘a bit of a problem’ versus ‘not a problem’. These questions were derived from questions used in the previous national survey of the lifestyle, attitudes and nutrition of people living in Ireland (SLAN) [76]. The variables were used as an interval/ordinal scale (‘0 to 9 neighbourhood problems’) in correlation and regression analyses.

### Statistical analysis

Analyses were conducted using SPSS 25 for Windows (IBM Corp., Armonk, New York, USA). Data were weighted by Ipsos MRBI and details about this process are described elsewhere [46]. Missing data were very low for all of the variables (< 5%). Data were examined for normality via histograms, and kurtosis and skew statistics. Distribution was not normal and could not be improved through transformation therefore sitting times in all of the domains investigated in the study were categorised as ordinal variables. Ordinal regression analyses were executed using sitting times in the three domains (occupation, leisure screen-time and transportation/leisure) and total sitting time. Means, standard deviations and medians were calculated for sitting times within the domains. Mean sitting times in terms of socio-economic classification were calculated to highlight how SB is distributed across the domains included in the study. Data on total sitting times were shown in terms of the various correlates included in the final models, to indicate the characteristics of those who engage in prolonged sitting. Multivariate ordinal regression analyses were executed to investigate associations between (i) biological and demographic; (ii) psychological, (iii) behavioural; (iv) social; and (v) physical environmental correlates with the dependent variables total sitting time, and the three domain-specific contexts of sitting. Separate binomial logistic regressions on cumulative dichotomous variables for each independent variable indicated that the assumption of proportional odds appeared tenable. Tests to see if the data met the assumption of collinearity indicated that multicollinearity was not a concern. *P*-values of less than 0.05 were considered statistically significant.

## Results

The mean age of the 7328 participants was 51 years (SD ± 17.8). Further descriptive characteristics are presented in Table 1 to address the first aim of the study. The median total sitting time of the sample was 450 min per day (IQR 290 min per day). The mean sitting time was 465.97 ± 193 min per day.

**Table 1:** Mean and standard deviation for total sitting in minutes/day, for intrapersonal, interpersonal, environment level influences

	N	Mean ± SD (Median)
Total sample	7328	465 ± 193 (450)
Age		
18–24	267	510 ± 179 (510)
25–34	467	480 ± 200 (470)
35–44	603	475 ± 201 (450)
45–54	447	477 ± 194 (480)
55–64	407	448 ± 187 (420)
65–74	203	392 ± 164 (370)
75–84	94	392 ± 165 (360)
85+	10	437 ± 140 (420)
Gender		
Female	1313	456 ± 193 (420)
Male	1185	477 ± 193 (480)
Long-standing illness		
No	1958	463 ± 194 (425)
Yes	538	477 ± 190 (450)
Education level		
</= Lower secondary	341	409 ± 185 (375)
Upper secondary	749	439 ± 187 (420)
Post-secondary course	442	458 ± 191 (420)
Bachelors or above	966	510 ± 193 (510)
Socio-economic classification		
Not classified	494	464 ± 185 (425)
Routine/manual	503	400 ± 174 (370)
Intermediate	633	460 ± 199 (420)
Higher managerial/professional	868	509 ± 193 (485)
Probable mental health problem		
No mental health problem	2310	463 ± 192 (433)
Probable mental health problem	188	506 ± 210 (480)
Physical activity levels		
Insufficient physical activity	1121	493 ± 200 (480)
Sufficient physical activity	1352	445 ± 185 (420)
Workplace activity		
Sitting	761	613 ± 167 (630)
Standing	281	366 ± 145 (360)
Mostly walking/moderate activity	553	370 ± 150 (360)
Mostly heavy labour/physically demanding	125	330 ± 140 (300)
Tobacco use		
No	2024	465 ± 193 (450)
Yes	474	472 ± 196 (450)
Audit-C		
1–4	951	465 ± 196 (420)
5–8	275	483 ± 197 (480)
> 6	772	490 ± 186 (480)
Marital status		
Married/civilpartnership	1366	456 ± 196 (420)
Not married/or in civil partnership	1132	478 ± 190 (480)
Caring Role		
No	2230	468 ± 194 (450)
Yes	268	448 ± 187 (420)
Neighbourhood attributes		
No Problem	893	456 ± 186 (420)
Some problems	1605	471 ± 197 (450)
Location		
Urban	1582	494 ± 191 (480)
Rural	916	418 ± 189 (390)

### Sitting time by domain specific context

**Fig. 2:**
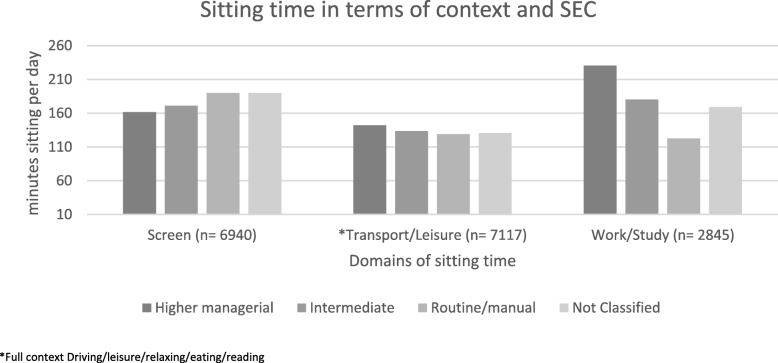
Mean domain specific sitting time by socio-economic classification

Figure 2 shows the domain specific average sitting time in minutes per day, by socio-economic classification. Mean sitting time was highest in work/study domain (195 ± 166), followed by screen-time sitting (184 ± 122) and transportation/leisure sitting (139 ± 95). Those in higher professional occupations had the longest sitting times per day in terms of both work/study sitting (230 ± 161) and transportation/leisure sitting time (142 ± 78), while those in routine/manual occupations had the longest leisure screen-time sitting (190 ± 107).

### Total sitting time

The strongest predictors of total sitting time were the intrapersonal factors of male gender, younger age, higher socio-economic classification and education levels, physical activity levels, having a long-term illness, and a probable mental health problem (Table 2). Having a caring role was associated with decreased sitting times. The environmental factors of living in an urban dwelling and increased neighbourhood ‘problems’ score were also associated with longer sitting times.

**Table 2:** Results of multivariate ordinal regression on the contribution of various correlates on total sitting

*N* = 1984	OR	CI	*p*-value
Gender			
Male	1.32	1.11–1.56	< 0.00
Female	1	Ref.	
Age^a^	0.99	0.98–0.99	< 0.00
Socio-economic status			
High	1.79	1.5–2.1	< 0.00
Low	1	Ref.	
Education			
High	1.43	1.21–1.68	< 0.00
Low	1	Ref.	
Long-term illness			
Yes	1.26	1.01–1.53	0.04
No	1	Ref.	
PMHP			
No	0.73	0.53–0.99	0.04
Yes	1	Ref.	
Physical activity			
Not sufficient	1.70	1.46–2.01	< 0.00
Sufficient	1	Ref.	
Tobacco			
No	0.20	0.93–1.38	0.20
Yes	1	Ref.	
Audit-C^a^	0.99	0.99–1.06	0.18
Marital status			
Married/Cohabiting	0.87	0.72–1.01	0.07
Single/Divorced/Widowed	1	Ref.	
Caring role			
No	1.30	1.01–1.67	0.04
Yes	1	Ref.	
Location			
Urban	2.03	1.72–2.4	< 0.00
Rural	1	Ref.	
Neighbourhood^a^	1.05	1.00–1.10	0.04

^a^Continuous variable

### Work/study sitting time

Table 3 outlines results of regression analysis investigating the association between multi-dimensional correlates and occupational sitting. The strongest predictors of occupational sitting time were male gender, younger age, higher socio-economic status and education levels, and low physical activity levels. Living in an urban location was associated with increased occupational sitting.

**Table 3:** Results of multivariate ordinal regression on the contribution of various correlates on occupational sitting

*N* = 2172	OR	CI	*p*-value
Gender			
Male	1.27	1.08–1.50	0.00
Female	1	Ref.	
Age^a^	0.98	.97–.98	< 0.00
Socio-economic status			
High	2.20	1.87–2.58	< 0.00
Low	1	Ref.	
Education			
High	1.57	1.33–1.84	< 0.00
Low	1	Ref.	
Long-term illness			
Yes	1.15	1.33–1.80	0.16
No	1	Ref.	
PMHP			
No	0.83	.613–1.13	0.23
Yes	1	Ref.	
Physical activity			
Not sufficient	1.55	1.33–1.80	< 0.00
Sufficient	1	Ref.	
Tobacco			
No	0.99	.95–1.04	0.68
Yes	1	Ref.	
Audit-C^a^	0.99	.96–1.03	0.68
Marital status			
Married/Cohabiting	0.96	.82–1.13	0.64
Single/Divorced/Widowed	1	Ref.	
Caring role			
No	1.20	.94–1.53	0.15
Yes	1	Ref.	
Location			
Urban	1.96	1.67–2.30	< 0.00
Rural	1	Ref.	
Neighbourhood^a^	0.99	.95–1.04	0.68

^a^Continuous variable

### Leisure screen-time sitting

The results of multivariate ordinal regression to investigate the association of multi-dimensional correlates on leisure screen-time sitting (Table 4) showed that male gender, increased age, lower socio-economic and education levels, physical and mental health problems, insufficient physical activity, smoking, and alcohol consumption were associated with increased SB in this domain. Being single/divorced/widowed, not having a caring role, and living in an urban location were associated with increased leisure screen-time sitting.

**Table 4:** Results of multivariate ordinal regression on the contribution of various correlates on leisure screen-time sitting

*N* = 5104	OR	CI	*p*-value
Gender			
Male	1.19	1.07–1.33	< 0.00
Female	1	Ref.	
Age^a^	1.02	1.01–1.02	< 0.00
Socio-economic status			
High	0.72	0.65–0.80	< 0.00
Low	1	Ref.	
Education			
High	0.78	0.70–0.87	< 0.00
Low	1	Ref.	
Long-term illness			
Yes	1.25	1.12–1.41	< 0.00
No	1	Ref.	
PMHP			
No	0.73	0.61–0.89	0.00
Yes	1	Ref.	
Physical activity			
Not sufficient	1.47	1.33–.162	< 0.00
Sufficient	1	Ref.	
Tobacco			
No	0.81	0.71–0.91	0.00
Yes	1	Ref.	
Audit-C^a^	1.05	1.03–1.10	< 0.00
Marital status			
Married/Cohabiting	0.79	0.72–0.88	< 0.00
Single/Divorced/Widowed	1	Ref.	
Caring role			
No	1.25	1.67–1.47	0.01
Yes	1	Ref.	
Location			
Urban	1.28	1.16–1.42	< 0.00
Rural	1	Ref.	
Neighbourhood^a^	1.02	0.10–1.05	0.11

^a^Continuous variable

### Transportation/leisure sitting time

The results of multivariate ordinal regression to investigate the association of socio-ecological correlates on transportation/leisure sitting (Table 5) showed that an increase in sitting time was associated with older age, higher socio-economic and education levels, physical health problems, insufficient physical activity, not smoking, and alcohol consumption. Being single/divorced/widowed, and higher neighbourhood ‘problem’ scores were associated with increased transportation/leisure sitting.

**Table 5:** Results of multivariate ordinal regression on the contribution of various correlates on sitting while driving/eating/reading/relaxing

*N* = 5258	OR	CI	*p*-value
Gender			
Male	1.00	0.90–1.12	0.97
Female	1	Ref.	
Age^a^	1.01	1.00–1.01	< 0.00
Socio-economic status			
High	1.17	1.01–1.30	0.00
Low	1	Ref.	
Education			
High	1.34	1.21–1.50	< 0.00
Low	1	Ref.	
Long-term illness			
Yes	1.33	1.18–1.49	< 0.00
No	1	Ref.	
PMHP			
No	1.10	0.90–1.32	0.39
Yes	1	Ref.	
Physical activity			
Not sufficient	1.12	1.02–2.41	0.02
Sufficient	1	Ref.	
Tobacco			
No	1.15	1.01–1.30	0.03
Yes	1	Ref.	
Audit-C^a^	1.03	1.01–1.05	0.01
Marital status			
Married/Cohabiting	0.90	0.80–0.98	0.02
Single/Divorced/Widowed	1	Ref.	
Caring role			
No	0.95	0.81–1.11	0.50
Yes	1	Ref.	
Location			
Urban	0.99	0.89–1.10	0.82
Rural	1	Ref.	
Neighbourhood^a^	1.04	1.01–1.07	0.00

^a^Continuous variable

## Discussion

The aim of this study was to investigate the factors that are associated with sedentary behaviour, as well as the domains in which this behaviour accumulates in an adult population-level cohort. These factors were informed by the SEM, which takes into account the different levels of correlates - intrapersonal, interpersonal and environmental factors [18].

The results indicated worryingly high levels of overall sitting of >7.5 h per day in the Irish population, given the all-cause mortality risk associated with sitting for > 7 h per day [16]. These levels are significantly higher than previous reports of 4 h per day of sitting time [35], and earlier preliminary findings reported in the 2015 Healthy Ireland Survey of 5.3 h per day [48]. A possible explanation could be differences in measures of sitting time used in the studies. The International Physical Activity Questionnaire short sitting question was used in both previous studies [35, 47], whereas total sitting time in the present study was calculated by summing the sitting times of the three domains measured. This may indicate a more accurate total sitting time as it captures SB separately for the most important daily contexts in which this behaviour occurs [18].

Highest sitting times accumulated for occupational sitting (> 3 h/day), in line with recently reported average daily occupational sitting [37], although somewhat lower than some previous studies [49–51]. The increase in desk-based occupations in recent decades has resulted in the workplace being a major contributor to sedentariness [52, 53]. Leisure screen-time sitting (2.5 h per day) in this study was higher than previous findings which included TV/tablet viewing within their screen-time measure [33]. Transportation/leisure sitting times (2 h/day) was in line with previous reports of this combination of these sitting domains [50].

Those with low levels of physical activity were the most sedentary in terms of all of the sitting contexts investigated in this study. This is in line with previous studies showing an inverse relationship between PA and SB [29, 54–57].

 In line with previous findings [34, 57] higher socio-economic classification and education levels were correlated with longer total sitting and occupational and transportation/leisure sitting times. The may be due to those with higher education attainment are more likely to be employed in more sedentary occupations. A recent review reported [19] that females who were older had higher total and leisure SB; however in our study males had significantly higher total, occupational and leisure screen-time SB. This is in line with studies that have found that total and occupational sitting was highest in males [34, 37]. Contrary to other reports that males had higher motorised travel sitting, we found no association with gender and the transportation/leisure context of SB.

De Cocker et al. [﻿48﻿] found that being a younger male was associated with increased occupational sitting while in front of a computer screen. We found less occupational and total sitting as age increased, whereas in the contexts of leisure screen-time and transportation/leisure SB, an increase in sitting times as age increased was found. Although higher education attainment was associated with greater transportation/leisure sitting time and occupational sitting, those with lower education attainment were positively associated with leisure screen-time sitting. This inverse relationship between screen-time sitting and education confirms previously reported findings [44, 58, 59], and TV viewing has been well established as being associated with lower socio-economic position [44, 59–61]. These nuances in terms of correlates and drivers of sedentary behaviour highlight the need to focus on the separate domains of sitting. Smoking was not found to be associated with increased total or occupational sitting in this study, however being a smoker was associated with increased screen-time sitting. Previous studies have reported an association with smoking and leisure screen-time sitting [62], while other studies have found correlations with smoking and total sitting times in studies of women [29, 62]. We also found that transportation/leisure sitting was associated with not smoking, which may be explained by the fact that smokers may leave their home to smoke outside.

Alcohol consumption was associated with increased leisure screen-time sitting and transportation/leisure sitting times. A recent review [19] found conflicting results regarding the relationship between alcohol consumption and SB, with three of the five studies included showing no association [29, 31, 64], while the remaining two studies found it to be positively associated with time spent sedentary in transportation [65] and to overall weekend sedentary time [63]. Relationships with alcohol are complex; this finding could be interpreted to mean that high risk drinkers sit more while in leisure screen-time viewing, possibly reflecting the recent culture of drinking more at home [66], while individuals who consume more alcohol in the transportation/leisure context may reflect a propensity for more sedentary leisure activities, such as reading or consuming alcohol while eating, further research is needed to investigate this.

Our study examined psychological factors associated with sedentary behaviour, and found that having a possible mental health problem was associated with increased total sitting times and leisure screen-time SB. This is in line with previous reports that individuals with major depressive disorders and comorbid depressive and anxiety disorders spend significantly more time in leisure SB while using the computer and TV viewing [65]. Recent reviews have found that SB is associated with increased risk of depression [67], and suggest positive associations between SB and anxiety risk [68]. The present study extends the research beyond screen-time SB and total SB by investigating associations between psychological wellbeing and other domains of SB such as occupational and leisure-time SB. Few studies have investigated psychological factors, and although we provide support for previous findings [69–71], outcome measures vary making direct comparisons difficult.

In terms of interpersonal factors, previous studies investigating TV SB reported that increased sitting was associated with being single [27], whereas Xie et al. [64] found TV viewing time to be higher in married people. Uijtdewilligen et al. [63] found that those who were married or living with a partner, were significantly less likely to be active compared to single women. We found an association between being married or in a civil partnership and lower leisure screen-time sitting and lower transport/leisure SB, in line with findings previously reported [28﻿].

Mixed results have been reported in previous studies investigating physical environment correlates depending on the SB context. Higher total sitting times have been reported in women in urban areas compared to those living in a rural location [29, 31], however increased SB associated with transportation was shown in rural-dwelling participants in two studies [57, 72]. The present study found that living in an urban location was associated with longer total, leisure screen-time, and occupational sitting times.

O’Donoghue et al. [19], in a systematic review, reported mixed results in terms of sedentary behaviour and various neighbourhood and community attributes. In a study including data from the US, Australia and Belgium, perceived aesthetics and proximity of destinations within participants’ neighbourhoods were associated with higher total sitting [28], while Compernolle et al. [73] reported no association between objectively measured neighbourhood attributes and total SB. Our study further investigated SB within various contexts and self-reported neighbourhood attributes and found a positive association between total- and transportation/leisure sitting times and neighbourhood factors. This adds evidence to the assertion that environmental characteristics related to perceived attributes of a neighbourhood may explain some of the variance in sedentary behaviour.

Our findings highlight the various and important correlates of sitting time in adults, as well as the physical and social contexts of where daily sitting time accumulates.

### Strengths

The strengths of this study include the large population-representative sample of the Healthy Ireland Survey, and the ability to assess correlates of sitting which can operate in distinct ways across different contexts; the results can therefore be generalised. This study is one of the few to investigate many potential factors associated with prolonged sitting time, including psychological influences, interpersonal factors, and neighbourhood factors that may influence SB. The novelty of this study lies in the various contexts of sitting included in this study that provides a comprehensive measure to calculate total sitting time. The inclusion of smartphone and tablet screen-time SB in the leisure screen-time SB measure captures more contemporary screen-time SB habits. Using a socio-ecological approach the study extends our knowledge on sedentary behaviour accumulated across multiple domains.

### Weaknesses

A limitation of this study is the use of self-report to assess sedentary behaviour. Self-report measures have limited validity due to issues with recall and social-desirability responses, however, there is consistency between self-report of SB and objective measures for most factors [19]. Transportation and leisure SB were combined in the present study, and although transportation SB may contribute the least to overall daily SB, it may be of benefit to investigate these domains separately. A further limitation in this study is the lack of differentiation between weekdays and weekend days in the Healthy Ireland Survey. Other characteristics of sitting such as prolonged bouts of sitting or sit/stand transitions were not captured. Body mass index and a breakdown of physical activity levels were not included in this dataset precluding important examination of these salient factors and their relationship with, and influence on, sedentary behaviour. Policy level factors were not available, which the SEM highlights as an important influence on behaviour. Finally, the cross-sectional design of this study provides information on factors associated or correlated with sedentary behaviour, thereby limiting causal inference and is subject to reverse causality.

### Implications of findings for policy and future research

This study establishes factors that may help understand sitting behaviour in an adult population, and importantly how these vary across specific domains of sitting, thereby providing valuable and relevant information for future development of effective interventions to reduce this damaging health behaviour. Males, with higher education and socio-economic classification levels, with a possible mental health problem, and those with insufficient levels of physical activity were the most likely to be sedentary. The domain in which most sitting was reported was occupational sitting, thus suggesting the workplace as a target setting for future interventions.

Subsequent research on SB will benefit from longitudinal designs that allow researchers to identify and predict determinants of sedentary behaviours, extrapolating them clearly from correlates. Surveillance using a combination of objective measures of sitting times including accelerometers/inclinometers, and contextual information obtained by subjective measures, would be preferable, however applying these on a large scale would be challenging. Homogeneity of outcome measures in future studies would be useful in terms of more in-depth analysis and provide more meaningful and useful conclusions. However, this evidence augments the Healthy Ireland survey findings and highlights the value of this data by enabling the application of the socioecological model to provide baseline information on important individual, social and environmental targets to incorporate into health promotion strategies and policy aimed at reducing sedentary behaviour.

## Conclusions

Sedentary behaviour remains high in the Irish population with the average sitting time reported at > 7.5 h per day. Workplace sitting contributed the most to total sitting time. Males, with sedentary occupations, in professional roles and in urban locations were most likely to be sedentary, therefore it is important to direct future policy and interventions to these groups.

## Data Availability

The data that support the findings of this study are available from the Department of Health but restrictions apply to the availability of these data, which were used under license for the current study, and so are not publicly available. The provision of access to the data rests with the Department of Health, and is available to researchers fulfilling assessment criteria.

## Abbreviations

AUDIT-C: Alcohol use disorders identification test consumption
CAPI: Computer assisted personal interview
IPAQ: International Physical Activity Questionnaire
METs: Metabolic equivalents
SB: Sedentary behaviour
SEM: Socio-ecological model
SF-36: Short form 36
